# Elicitation of resistance and associated defense responses in *Trichoderma hamatum* induced protection against pearl millet downy mildew pathogen

**DOI:** 10.1038/srep43991

**Published:** 2017-03-21

**Authors:** Chandra Nayaka Siddaiah, Niranjan Raj Satyanarayana, Venkataramana Mudili, Vijai Kumar Gupta, Selvakumar Gurunathan, Shobith Rangappa, Shekar Shetty Huntrike, Rakesh Kumar Srivastava

**Affiliations:** 1 Department of Studies in Biotechnology, University of Mysore, Manasagangotri, Mysore, 570006, Karnataka, India; 2 Department of Studies in Microbiology, Karnataka State Open University, Mukthagangotri, Mysore, 570006, Karnataka, India; 3 Microbiology Division, DRDO-BU-Centre for Life sciences, Bharathiar University Campus, Coimbatore, 641046, Tamil Nadu, India; 4 Discipline of Biochemistry, National University of Ireland Galway, Galway, Ireland; 5 Frontier Research Center for Post-Genome Science and Technology, Hokkaido University, Sapporo 060-0808, Japan; 6 International Crops Research Institute for the Semi-Arid Tropics (ICRISAT), Patancheru, 502324, Telangana, India

## Abstract

Endophytic *Trichoderma hamatum* UoM 13 isolated from pearl millet roots was evaluated for its efficiency to suppress downy mildew disease. Under laboratory conditions, *T. hamatum* seed treatment significantly enhanced pearl millet seed germination and seedling vigor. *T. hamatum* seed treatment resulted in systemic and durable immunity against pearl millet downy mildew disease under greenhouse and field conditions. *T. hamatum* treated seedlings responded to downy mildew infection with high lignification and callose deposition. Analysis of defense enzymes showed that *T. hamatum* treatment significantly enhanced the activities of glucanase, peroxidase, phenylalanine ammonia-lyase, and polyphenol oxidase in comparison to untreated control. RT-PCR analysis revealed differentially expressed transcripts of the defense enzymes and PR-proteins in treated, untreated, and checks, wherein PR-1, PR-5, and cell wall defense HRGPs were significantly over expressed in treated seedlings as against their lower expression in controls. *T. hamatum* treatment significantly stimulated endogenous salicylic acid (SA) levels and significantly upregulated important SA biosynthesis gene isochorismate synthase. The results indicated that *T. hamatum* UoM13 treatment induces resistance corresponding to significant over expression of endogenous SA, important defense enzymes, PR-proteins, and HRGPs, suggesting that SA biosynthetic pathway is involved in pearl millet for mounting systemic immunity against downy mildew pathogen.

Pearl millet (*Pennisetum glaucum* (L.) R. Br.), occupies a unique position among cereal crops because of its ability to grow under poor environments and provide nutritious food, feed, fodder, and livelihood support. It is a staple food for millions of people around the world. It is often cultivated under extremely harsh conditions of frequent drought, high temperature, low and erratic rainfall in infertile soils having poor water holding capacity. It is currently grown in an area of over 28 million hectares worldwide, upon which approximately 500 million people depend on for their survival. In India, it is grown in an area of 7.95 million hectares with an annual production of 8.9 million tons^1^. Since growth in cultivated areas is unlikely to contribute much to future production, the burden of meeting increased demand for pearl millet rests on improvements in crop yields.

Downy mildew disease caused by the biotrophic oomycete *Sclerospora graminicola* (Sacc.) Schroet, is a major constraint for pearl millet production, causing exhaustive losses up to 80%^1^,^2^. Management of pearl millet downy mildew is achieved by cultural practices, use of resistant cultivars, chemical, and biological control, but each with its own shortcomings^3^. High variation in genetics and virulence among isolates of *S. graminicola* results in regular breakdown of resistance^3^. Currently recommended chemicals like metalaxyl are hazardous and also not easily accessed by the farmers. Lack of effective mass production and delivery methods have limited the use of biological control methods. Hence, there is always a search for an alternative to the use of chemicals, which can offer effective control and at the same time be economical, safe and eco-friendly.

*Trichoderma* spp. are free-living opportunistic, avirulent plant symbiotic or parasitic fungi inhabiting soil and root ecosystems. *Trichoderma* occurs as endophytes often colonizing the roots and penetrate a few cell layers beyond the epidermis^4^. Such colonization of roots by *Trichoderma* often results in better uptake of nutrients, increased root growth and development, enhanced productivity and, improved tolerance to various stresses including diseases. *Trichoderma* produces a wide spectrum of secondary metabolites that are known to serve as triggers of systemic resistance against a broad range of plant pathogens^5^. There are numerous reports of various *Trichoderma* spp. induced resistance against different plant pathogens. Root colonization by different *Trichoderma* spp. like *T. asperellum, T. hamatum, T. harzianum* and *T. virens* have triggered systemic acquired resistance (SAR) against different diseases^4^. Particularly, *Trichoderma hamatum* has been efficient in triggering SAR in many host-pathosystems. *Trichoderma hamatum* T382 effectively induced resistance against *Botrytis cinerea* of *Arabidopsis*^6^, and *Xanthomonas vesicatoria* in tomato^7^. *Trichoderma hamatum* GD12 elicited resistance against *Magnaporthe oryzae* in rice^8^*, Sclerotinia sclerotiorum* and *Rhizoctonia solani* in lettuce^9^,^10^.

Induction of resistance by several elicitors against a range of pathogens has been shown to be mediated by the plant hormone salicylic acid (SA) accumulation^11^. SA is also vital for the expression of pathogenesis-related (PR) genes and the synthesis of defensive compounds including lignin, callose, and various defense enzymes which are associated with both local and systemic acquired resistance^12^. SA biosynthesis significantly enhanced upon tobacco mosaic virus infection in tobacco and the *Pseudomonas syringae* infection in *Arabidopsis* resulting in increased host resistance against these pathogens^13^,^14^.

The primary objective of the present study was to evaluate if endophytic *T. hamatum* strain UoM 13 isolated from pearl millet host plants is efficient to elicit systemic immunity against pearl millet downy mildew disease. Previous studies from our laboratory demonstrated the role of important defense proteins, like PR1, PR5, hydroxyproline-rich glycoproteins (HRGPs) and defense enzymes such as peroxidase (POX), β-1,3-glucanase, phenylalanine ammonia-lyase (PAL), and polyphenol oxidase (PPO), in the pearl millet–*S. graminicola* interaction. Therefore, the role of some of the important defense enzymes and PR proteins involved in *T. hamatum* induced SAR in pearl millet were evaluated. Since enhanced production of antimicrobial enzymes and pathogenesis-related (PR) proteins are induced by the SA signaling during induction of resistance, the present study also aimed to analyze the role of endogenous SA accumulation and SA biosynthetic genes.

## Results

### Molecular identification of *T. hamatum* UoM 13

**Figure 1:**
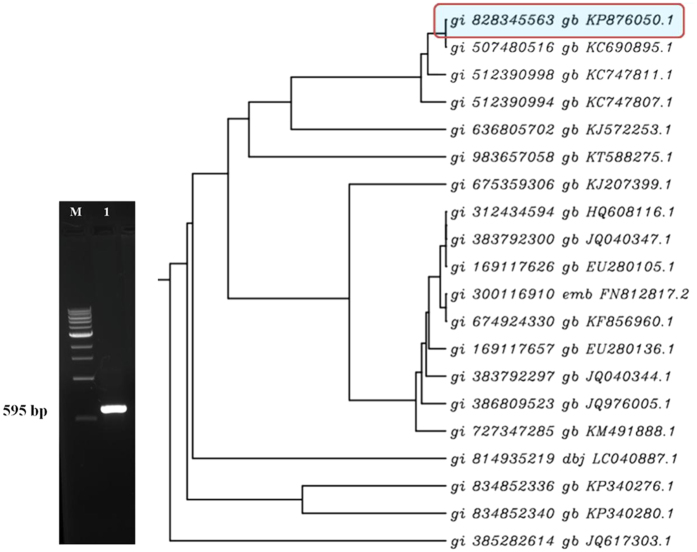
PCR amplification of *T. hamatum* with ITS1/ITS4 universal primer and Phylogenetic relationships of endophytic *T. hamatum* UoM 13 strains with other *Trichoderma* spp. based on ITS rDNA sequences. The tree was constructed using the Clustal w 2.1 multiple sequence alignment programs.

The ITS1 and ITS4 primer amplified ~560 bp fragment from the entire genomic DNA isolated from a pure culture of *T. hamatum* UoM 13 and no non-specific bands were observed. PCR products were sequenced by the dideoxynucleotide method using the Big Dye Terminator ver. 3.0 Kit (Chromos biotech) from both strands. The sequence homology was made by using BLAST (Basic Local Alignment Search Tool) program and multiple sequence alignment was with related *Trichoderma* species was performed using Multalin server by using CLUSTAL 2.1 software (Fig. 1). The sequence information was deposited in NCBI database and accession number was obtained (KP 876050).

### Effect of endophytic *T. hamataum* UoM 13 on seed germination and seedling vigor of pearl millet under laboratory conditions

In comparison with the distilled water treated control, all the treatments significantly enhanced seed germination and seedling vigor, but the rate of enhancement varied with treatments. *T. hamataum* UoM 13, chitosan and Apron treatments recorded 94, 90, and 88% germination as against 83% in distilled water treatment. *T. hamatum* UoM 13 recorded maximum seedling vigor among all the treatments and control. *T. hamatum* UoM 13 seedlings recorded 1928 seedling vigor which was significantly higher than chitosan, Apron and control treatments, which showed 1907, 1896 and 1872 seedling vigor respectively (Table 1).

### Screening of endophytic *T. hamataum* UoM 13 for potential to elicit resistance against downy mildew under greenhouse and field conditions

#### Greenhouse studies

In general, all the tested treatments protected pearl millet against downy mildew disease, but the degree of protection offered varied considerably. Each treatment resulted in a significant reduction in the number of plants with downy mildew disease in comparison with the distilled water control. Among the inducer treatments, highest protection resulted from chitosan seed treatment which recorded 17.4% downy mildew as against 96% downy mildew in control plants. *Trichoderma hamatum* UoM 13 seed treatment resulted in 18.2% downy mildew which was not significantly different from chitosan treatment (Supplementary Figure 1). Treatment with fungicide control Apron recorded the least downy mildew disease of 8.8% which was significantly less compared to the inducer treatments and control (Table 1).

#### Demonstration of the nature of resistance induction by T. hamatum UoM 13

**Figure 2:**
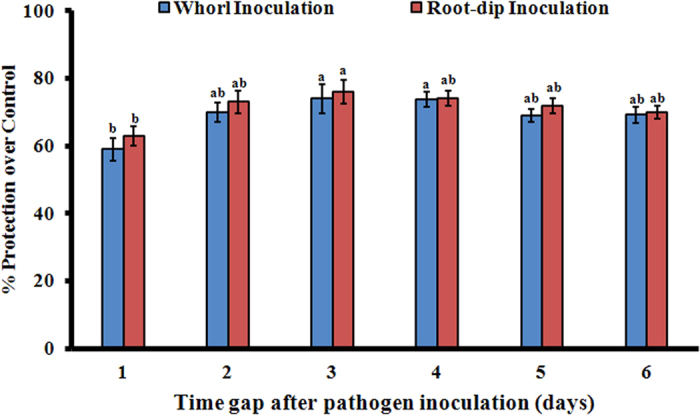
Demonstrations of systemic nature of resistance induction by *T. hamatum* UoM 13 by spatio-temporal separation of the inducer and pathogen inoculation. Emerging pearl millet seedligns raised from 7042S seeds treated with *T. hamatum* UOM 13 were challenge-inoculated with the zoospore suspension of *S. graminicola* by adding 4–5 drops (0.5 ml) to the leaf whorl of each plant at intervals of 1, 2, 3, 4, 5 and 6 days between the seedling emergence and pathogen inoculation in different sets of plants. Bars indicat the standard error as indicated by Tukey’s HSD (P = 0.05).

*Trichoderma hamatum* UoM 13 when tested further for the nature of resistance induced by following the spatial and temporal separation method it was found to be systemic. Initially, the isolate protected the pearl millet plants up to 61% when the time gap was 1 day. The protection percentage was raised to 70% on the second day and 74% on the third day. This resistance was consistently maintained throughout the experimental period thus indicating that a minimum 3 days were required for the total resistance build up. The trend was similar in the second set of experiments where the inducer treatment was given as root dip inoculation. Initially, at 1-day gap, the protection offered was 65%. This shot up to 73% and 76% on the second and third-day gap, respectively. This protection percentage was sustained throughout the experimental period (Fig. 2).

#### Field studies

Under field conditions, a significant reduction in downy mildew disease was observed in the test treatments when compared to the distilled water treated control rows. The highest protection resulted from chitosan seed treatment which recorded 12.5% downy mildew as against 92.5% downy mildew in control plants. *Trichoderma hamatum* seed treatment resulted in 15.6% downy mildew and was not significantly different from chitosan treatment. However, Apron treatment recorded the least downy mildew disease of 5.1% which was significantly less compared among all the test treatments (Table 1).

### Time course analysis of lignification

**Figure 3:**
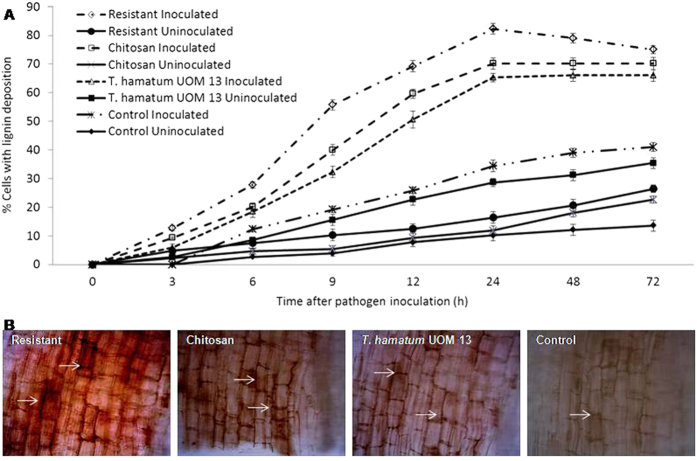
(**A**) Percentage of cells showing lignification in pearl millet seedlings at different time intervals with (inoculated) or without (uninoculated) *Sclerospora graminicola* inoculation. Resistant – Seedlings of downy mildew resistant cultivar, Chitosan-Seedlings treated with Chitosan, *T. hamatum* UOM 13-Seedlings treated with the endophyte *T. hamatum* UOM 13, Control: Seedlings of downy mildew susceptible cultivar. Vertical bars indicate standard error. Results are average of three independent experiments with four replicates of 25 seedlings each. (**B**) Light microscopic (bright field) pictures showing the deposition of lignin in epidermal peelings from the coleoptile region pearl millet seedlings 24 h after inoculation with *Sclerospora graminicola*. Lignification detected by phloroglucinol-HCL staining method.

Lignification was observed as reddish brown depositions on the cell walls, along the cell wall and in region of papilla formation (Fig. 3). At 0 hours after inoculation (hai) no lignification was observed in any of the test seedlings with or without pathogen inoculation. However, after 3 hai up to 24 hai there was a gradual increase in lignification which plateaued thereafter. At 24 hai, resistant, chitosan and *T. hamatum* UoM treated seedlings showed 82, 70 and 68% lignification as against the control seedlings which showed only 38% lignification. At 24 hai, lignification in *T. hamatum* UoM treated seedlings was 56.2% more than the control seedlings at the same time point. In the uninoculated samples, lignin accumulation was significantly lower than that of the pathogen-inoculated samples. However, among these uninoculated samples, highest lignin accumulation of 38.4% was observed in *T. hamatum* UoM 13 treated seedlings, which was1.45, 1.69 and 2.82 folds higher than that of the resistant, chitosan treated, and untreated control, respectively.

Lignification when evaluated objectively using the four-point scale, at 0 hai no lignification was observed in any seedlings with or without pathogen inoculation. The intensity of lignification gradually increased from 3 hai up to 24 hai. In the inoculated samples, percentage of seedlings with high localization was observed in resistant seedlings followed by chitosan and *T. hamatum* UoM treated seedlings. On the contrary, the control seedlings showed maximum cells with no localization and only a small percentage of cells with high lignification (Supplementary Figure 2). Among the uninoculated samples, only moderate amount of lignification was observed, and *T. hamatum* UoM 13 treated seedlings showed higher lignification compared to the other uninoculated categories of seedlings.

### Time course analysis of callose deposition

**Figure 4:**
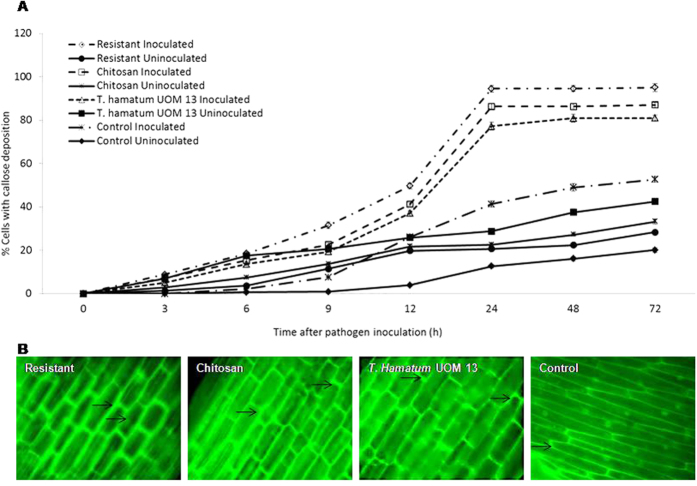
(**A**) Percentage of cells showing callose deposition in pearl millet seedlings at different time intervals with (inoculated) or without (uninoculated) *Sclerospora graminicola* inoculation. Resistant – Seedlings of downy mildew resistant cultivar, Chitosan-Seedlings treated with Chitosan, *T. hamatum* UOM 13-Seedlings treated with the endophyte *T. hamatum* UOM 13, Control: Seedlings of downy mildew susceptible cultivar. Vertical bars indicate standard error. Results are average of three independent experiments with four replicates of 25 seedlings each. (**B**) Light microscopic (fluorescence) pictures showing the deposition of callose in epidermal peelings from the coleoptile region pearl millet seedlings 24 h after inoculation with *Sclerospora graminicola*. Callose deposition detected by aniline blue staining method.

Callose depositions were observed as bright greenish yellow fluorescence along the cell wall and in the region of papilla formation (Fig. 4). At 0 hai no callose deposition was observed in any of the test seedlings with or without pathogen inoculation. However, after 3 hai up to 24 hai there was a gradual increase in callose deposition which plateaued thereafter. At 24 hai, resistant, chitosan and *T. hamatum* UoM treated seedlings showed 94, 86 and 77% callose deposition as against the control seedlings which showed only 48% callose deposition. At 24 hai callose deposition in *T. hamatum* UoM treated seedlings was 62.3% more than the control seedlings at the same time point. The intensity of callose deposition was comparatively lower in uninoculated samples compared to the pathogen-inoculated samples. Among the uninoculated samples, maximum callose deposition of 46.5% was noted in *T. hamatum* UoM 13 treated seedlings which were 1.64, 1.40 and 2.10 folds higher than resistant, chitosan treated and untreated control seedlings respectively.

Callose deposition when evaluated objectively using the four-point scale, at 0 hai no depositions was observed in any seedlings with or without pathogen inoculation. Following pathogen inoculation, the intensity of callose deposition gradually increased from 3 hai up to 24 hai. Percentage of seedlings with high deposition was observed in resistant seedlings followed by chitosan and *T. hamatum* UoM treated seedlings. Control seedlings showed maximum cells with no callose deposition and only a small percentage of cells with high callose deposition (Supplementary Figure 3). Compared to the pathogen inoculated samples, callose deposition in uninoculated samples were lesser, and in *T. hamatum* UoM 13 treated samples, callose deposition was higher than that of all other uninoculated samples.

## Biochemical Studies

### Enzyme activities

#### Phenylalanine ammonia lyase activity

**Figure 5:**
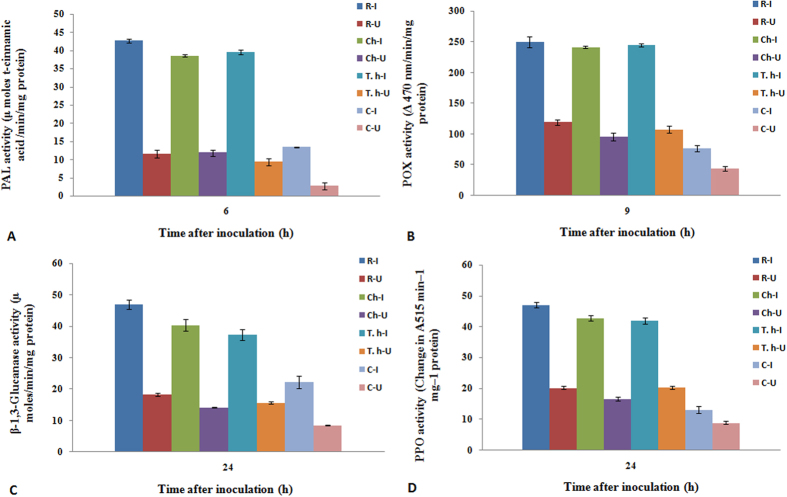
Pattern of accumulation of defense-related enzymes in two-day old pearl millet seedlings with (inoculated) or without (uninoculated) *Sclerospora graminicola* inoculation. Resistant – Seedlings of downy mildew resistant cultivar, Chitosan-Seedlings treated with Chitosan, *T. hamatum* UOM 13-Seedlings treated with the endophyte *T. hamatum* UOM 13, Control: Seedlings of downy mildew susceptible cultivar. (**A**) Phenylalanine ammonia lyase was determined as activity was determined as the amount of t-cinnamic acid formed from L-Phenylalanine per mg of protein per min measured spectrophotometrically at a wavelength of 290 nm (**B**) Peroxidase activity determined as the increase in absorbance recorded 470 nm. POX activity is expressed in terms of the change in A_470_ min^−1^ mg^−1^ protein (**C**) β-1,3 Glucanase activity was expressed in terms of μ moles per mg protein per min (**D**) Polyphenol oxidase determined as increase in absorbance at 420 nm was recorded for 1 min. The results are expressed as the change in A per min per mg protein Data of enzyme activity are means ± SE of three different experiments. The values were the means of three replicates of three different experiments. Bars indicate standard errors; means with different superscripts are significantly different, as shown by Tukey’s HSD test (P = 0.05).

Constitutive PAL activity was observed in all the test and control seedlings with or without pathogen inoculation which varied significantly with treatments. However, after pathogen inoculation there was significant increase in the enzyme activity which peaked at 6 hai and gradually decreased thereafter (Supplementary Figure 4a). Highest PAL activities were recorded in resistant seedlings which showed 42.6 units at 6 hai. In *T. hamatum* treated seedlings highest PAL activity of 39.6, which were 1.02 and 2.9 folds higher than the chitosan treatment and control respectively (Fig. 5A). At all tested time points, PAL activity was higher in pathogen-inoculated seedlings compared to the uninoculated seedlings. However, among the uninoculated seedlings, at 6 h after harvesting *T. hamatum* UoM treated seedlings showed 4.83 folds higher PAL activity than the untreated uninoculated control.

#### Peroxidase activity

Constitutive POX activity was recorded in all the test and control seedlings with or without pathogen inoculation. After pathogen inoculation, high POX activity was noticed in all categories of seedlings after inoculation of *S. graminicola*. POX activity peaked at 9 hai in all categories of seedlings (Supplementary Figure 4b). Highest POX activity of 249.8 units was recorded in resistant seedlings followed by 241.1 units recorded in chitosan treated seedlings at 9 hai. Treatment with *T. hamatum* UoM 13 also recorded significantly high POX activity of 244.9 units after 9 hai which was 3.2 folds higher than that of the untreated control which recorded 76.5 units POX activity after 9 hai (Fig. 5B). Time course pattern of POX activity in uninoculated samples were similar to the pathogen inoculated samples, however, the amount of enzyme activity was significantly lower in uninoculated samples. Among the uninoculated samples, high POX activity was observed in resistant samples. *T. hamatum* UoM 13 treated seedlings without inoculation showed 105.65 units POX activity, which was 1.11 and 2.48 folds higher than that of chitosan treated and untreated uninoculated controls respectively.

#### β-1,3-Glucanase activity

Constitutive β-1, 3-glucanase activity was observed in all the categories of seedlings with or without pathogen inoculation which varied significantly with treatments. However, after pathogen inoculation there was significant increase in the enzyme activities (Supplementary Figure 4c). In all test seedlings glucanase activity peaked at 24 hai. Maximum glucanase activity was recorded in resistant seedlings which showed 46.8 units at 24 hai. Among the treated seedlings, highest β-1,3-glucanase activity was recorded in chitosan treatment which showed 40.3 units followed by *T. hamatum* UoM 13 treatment which showed 37.3 units at 24 hai which were 1.8 and 1.6 folds higher respectively than the control seedlings which recorded 22.2 units β-1,3-glucanase activities at the same time point (Fig. 5C). Among the uninoculated samples maximum glucanase activity was observed in resistant seedlings. Uninoculated *T. hamatum* UoM 13 treated seedlings showed 15.65 glucanase activity which was 1.1 and 1.85 folds higher than that of chitosan treated and untreated controls, respectively.

#### Polyphenol oxidase activity

Constitutive PPO activity was observed in all categories of seedlings with or without pathogen inoculation which rapidly increased after pathogen inoculation. PPO activity was found to gradually increase from 3 hai and peaked at 24 hai (Supplementary Figure 4d). In resistant seedlings maximum PPO activity of 47.02 units was recorded at 24haiwhile chitosan treated seedlings at the same time point recorded 42.6 units PPO activity. At 24 hai *T. hamatum* UoM 13 treated seedlings recorded 41.9 units PPO activity which was 3.2 folds higher than that of the control seedlings which showed 13.09 units PPO activity at the same time point (Fig. 5D). PPO activity in uninoculated samples was highest in *T. hamatum* UoM treated seedlings, which was at par with resistant seedlings, and 1.22 and 2.57 folds higher than that of chitosan treated and untreated controls, respectively.

#### Analysis of hydroxyproline-rich glycoproteins

**Figure 6:**
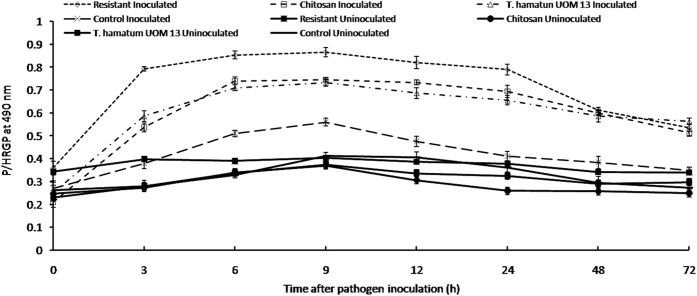
Temporal pattern of accumulation of Hydroxyproline-rich glycoproteins in two-day old pearl millet seedlings harvested at 0, 3, 6, 9, 12, 24, 48 and 72 h with (inoculated) or without (uninoculated) *Sclerospora graminicola* inoculation. Hyp content was expressed as μg Hyp mg^−1^ cell wall (dry weight). Resistant – Seedlings of downy mildew resistant cultivar, Chitosan-Seedlings treated with Chitosan, *T. hamatum* UOM 13-Seedlings treated with the endophyte *T. hamatum* UOM 13, Control: Seedlings of downy mildew susceptible cultivar. Data of enzyme activity are means ± SE of three different experiments. The values were the means of three replicates of three different experiments. Bars indicate standard errors; means with different superscripts are significantly different, as shown by Tukey’s HSD test (P = 0.05).

Constitutive HRGPs activity was recorded in all categories of seedlings with or without pathogen inoculation. After inoculation of the pathogen, HRGP activity gradually increased and peaked at 9 hai (Fig. 6). At 9 hai, HRGP activity was highest in resistant seedlings which recorded 0.866 activity followed by chitosan treatment which recorded 0.746 activity. *T. hamataum* UoM 13 treatment after 9 hai showed 0.733 activity, which was 1.3 folds higher than the untreated control seedlings, which recorded 0.56 activity at the same time point. Among the uninoculated samples, high HRGPs activity was observed in the resistant seedlings. *T. hamatum* UoM 13 treated seedlings showed 0.368 HRGPs activity, which was 1.03 and 0.88 folds higher than that of chitosan treated and untreated controls, respectively.

### Analysis of endogenous salicylic acid accumulation

**Figure 7:**
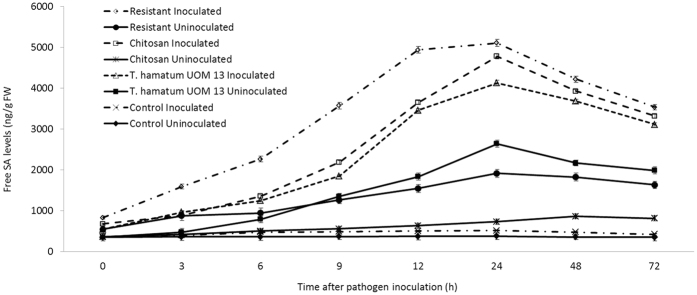
Temporal pattern of accumulation of salicylic acid in two-day old pearl millet seedlings harvested at 0, 3, 6, 9, 12, 24, 48 and 72 h with (inoculated) or without (uninoculated) *Sclerospora graminicola* inoculation. Quantification of SA was done by wavelength scan identification of elicitation wavelength of 312 nm and an emission wavelength of 415 nm Resistant – Seedlings of downy mildew resistant cultivar, Chitosan-Seedlings treated with Chitosan, *T. hamatum* UOM 13-Seedlings treated with the endophyte *T. hamatum* UOM 13, Control: Seedlings of downy mildew susceptible cultivar. Data of enzyme activity are means ± SE of three different experiments. The values were the means of three replicates of three different experiments. Bars indicate standard errors; means with different superscripts are significantly different, as shown by Tukey’s HSD test (P = 0.05).

At constitutive level SA was present in all the categories of seedlings with or without pathogen inoculation; however, in resistant seedlings the level of SA was slightly higher than others. In all categories of seedlings, following pathogen inoculation, SA accumulation peaked at 24 hai. At 24 hai SA accumulation was maximum in resistant seedlings which was 1.05, 1.23 and 9.9 folds higher than chitosan treated, *T. hamatum* UoM treated and control seedlings respectively. The SA accumulation in *T. hamatum* UoM 13 treated seedlings at 24 hai was 8.0 folds higher than that of control seedlings. After 24 hai there was a gradual decrease in SA accumulation in all categories of seedlings (Fig. 7). The amount of SA accumulation in uninoculated seedlings was considerably lower than inoculated seedlings in all samples. *T. hamatum* UoM 13 treated seedlings showed 1.37, 3.6 and 7.01 folds higher SA accumulation than the uninoculated resistant, chitosan treated, and untreated controls, respectively.

### Gene expression studies

#### Quantitative real-time PCR analysis (qPCR)

**Figure 8:**
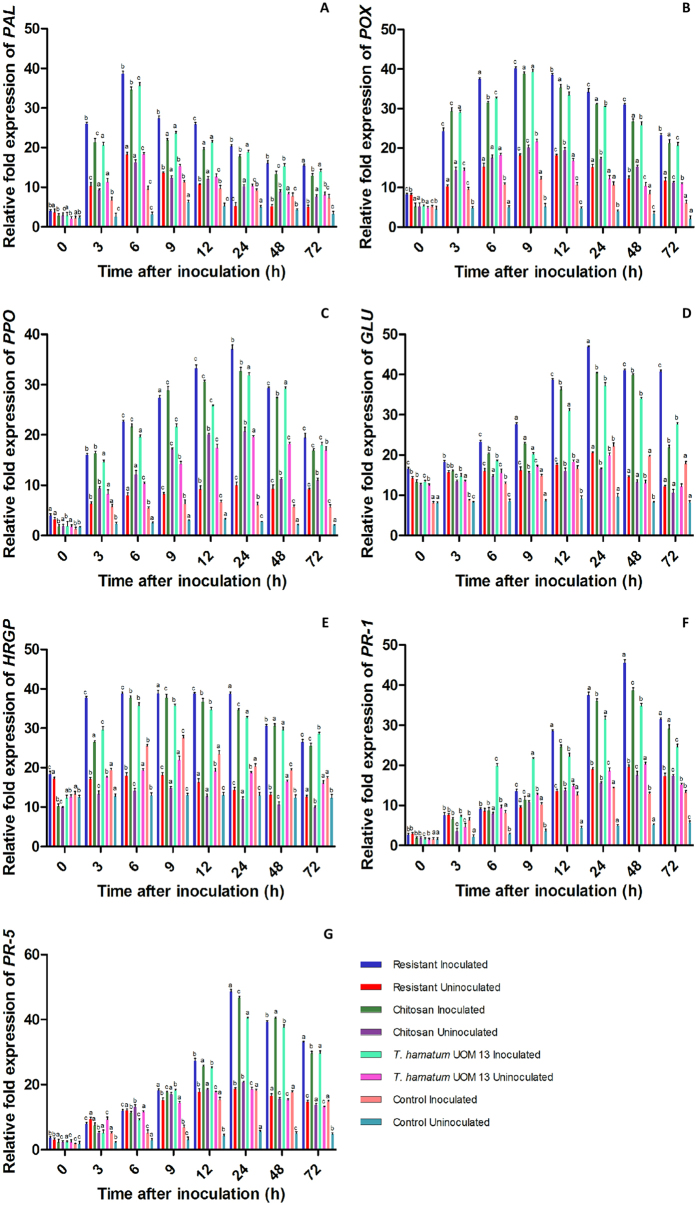
qRT-PCR determined relative expression of genes of various defense enzymes in two-day-old pearl millet seedlings with (I) or without (U) *Sclerospora graminicola* inoculation harvested 0, 3, 6, 9, 12, 24, 48, and 72** **h. Resistant – Seedlings of downy mildew resistant cultivar, Chitosan-Seedlings treated with Chitosan, *T. hamatum* UOM 13-Seedlings treated with the endophyte *T. hamatum* UOM 13, Control: Seedlings of downy mildew susceptible cultivar. (**A**) Phenylalanine ammonia lyase (**B**) Peroxidase (**C**) β-1,3 Glucanase (**D**) Polyphenol oxidase (**E**) PR-1 (**F**) PR-5 and (**G**) hydroxyproline-rich glycoprotein. Expression levels were measured by qPCR and normalized to the constitutive PP2A gene. Values are means of a single experiment carried out in triplicate. The bars indicate ± SE and the data were analyzed by one-way ANOVA followed by Tukey’s test and p-value < or = 0.05 was significant compared with control and <0.01 significant with treated control.

Real-time PCR analysis was carried out to investigate the priming effect of *T. hamatum* UoM13 on mRNA expression of defense-related gene expression in comparison with the resistant and chitosan checks along with untreated control seedlings with or without pathogen inoculation (Fig. 8A–G). The resistant, chitosan treated and *T. hamatum* UoM 13 treated seedlings after challenge inoculation with the downy mildew pathogen showed rapid and significantly enhanced expression of PAL, POX, PPO, glucanase, HRGPs, PR1, and PR5 genes in comparison with the untreated control seedlings. Constitutive levels of gene expression were observed for all the tested genes in all categories of seedlings, which gradually increased after pathogen inoculation. However the expression level was higher in resistant seedlings.

After inoculation with the downy mildew pathogen PAL expression gradually increased and peaked at 6 hai there was a drastic increase in PAL gene expression at 3 hai and it peaked at 6 hai which decreased thereafter. At this point PAL expression in *T. hamatum* UoM 13 treated seedlings was 3.65 folds higher than the control seedlings. In uninoculated samples of *T. hamatum* UoM 13 treated seedlings, maximum PAL expression was observed at 6 h which was at par with resistant seedlings, and 1.11 and 5.84 folds higher than chitosan treated and untreated controls, respectively (Fig. 8A).

POX gene expression gradually increased and maximum POX transcript level was recorded at 9 hai which decreased thereafter. At 9 hai POX expression in *T. hamatum* UoM 13 seedlings was 3.42 folds higher in comparison to the control seedlings. Without pathogen inoculation, maximum POX expression was observed at 9 h and *T. hamatum* UoM 13 treated seedlings showed highest expression which was 1.19, 1.07 and 4.22 folds higher than resistant, chitosan treated and untreated control, respectively (Fig. 8B).

PPO gene expression gradually increased following pathogen inoculation and the expression peaked at 24 hai. In *T. hamatum* UoM 13 treated seedlings PPO expression at 24 hai was 5.31 folds higher than the control seedlings. After pathogen inoculation and up to 24 hai PPO expression in *T. hamatum* UoM 13 treated seedlings was lower than that of resistant and chitosan treated checks, however, after this time point, PPO expression was higher in *T. hamatum* UoM 13 treatment in comparison with chitosan treatment and control. PPO expression in uninoculated *T. hamatum* UoM 13 seedlings was at par with uninoculated chitosan treated seedlings, and 1.96 and 7.17 folds higher than resistant and untreated controls, respectively (Fig. 8C).

Glucanase expression gradually increased following pathogen inoculation and maximum expression was recorded at 24 hai glucanase expression in *T. hamatum* UoM 13 treated seedlings at 24 hai was 1.85 folds higher than the control seedlings. It was observed that at 72 hai glucanase expression was higher in *T. hamatum* UoM 13 treated seedlings compared to both resistant and chitosan treated seedlings. Uninoculated samples recorded significantly lower glucanase expression compared to the inoculated samples, and at 24 h *T. hamatum* UoM 13 treated seedlings showed glucanase expression on par with resistant seedlings, 1.2 and 2.0 folds higher than chitosan treated and untreated control respectively (Fig. 8D).

HRGPs gene expression gradually increased after pathogen inoculation and the transcript accumulation peaked at 9 hai. HRGPs gene expression at 9 hai hai in *T. hamatum* UoM 13 treated seedlings was 1.71 folds higher compared to the control seedlings. It was observed that in resistant seedlings HRGPs gene expression was maintained at the same level from 3 to 24 hai. In *T. hamatum* UoM 13 treated seedlings HRGPs gene expression was same from 6 to 12 hai which decreased thereafter. However, at 72 hai HRGPs gene expression in *T. hamatum* UoM 13 treated seedlings was significantly higher than that of resistant and chitosan checks. The pattern of HRGPs expression in uninoculated samples were similar to the inoculated samples however, the intensity was significantly lesser than that of the inoculated samples. Uninoculated *T. hamatum* UoM 13 treated seedlings showed maximum HRGP expression and at 9 h the expression was 1.21, 1.11 and 1.68 folds higher than resistant, chitosan treated and untreated control respectively (Fig. 8E).

PR-1 gene expression gradually increased after pathogen inoculation and the expression level peaked at 48 hai. *Trichoderma hamatum* UoM 13 treated seedlings at 48 hai recorded 3.55 fold higher PR-1 gene expression than that of control seedlings. It was observed that in *T. hamatum* UoM 13 treated seedlings recorded higher PR-1 expression that that of both resistant and chitosan checks up to 9 hai and thereafter its expression was lower than that of resistant and chitosan checks. Temporal expression pattern of PR-1 was similar in both uninoculated and inoculated samples; however, in uninoculated samples the intensity was significantly lower. At 48 h, uninoculated *T. hamatum* UoM 13 treated seedlings showed 3.90 folds higher PR-1 expression than the untreated control (Fig. 8F).

PR-5 gene expression gradually increased following pathogen inoculation and maximum expression was recorded at 24 hai. In *T. hamatum* UoM 13 treated seedlings PR-5 expression was 1.84 folds higher than that of control seedlings. PR-5 gene expression was higher in resistant and chitosan checks at all time points except at 9 hai in comparison to the *T. hamatum* UoM 13 treated and control seedlings. PR-5 genes expression in uninoculated samples showed lower expression than inoculated samples for all categories of test seedlings. Uninoculated *T. hamatum* UoM 13 treated seedlings showed 3.28 folds higher PR-5 expression than the untreated control (Fig. 8G).

#### SA synthesis gene expression

**Figure 9:**
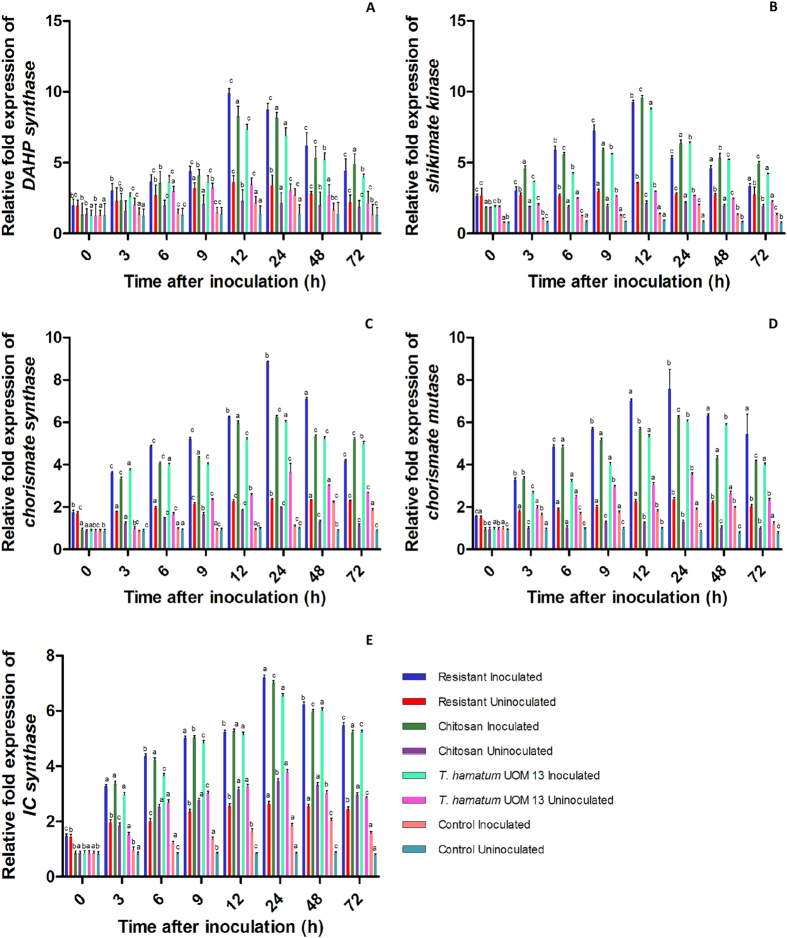
qRT-PCR determined relative expression SA biosynthesis genes in two-day-old pearl millet seedlings with (I) or without (U) *Sclerospora graminicola* inoculation harvested 0, 3, 6, 9, 12, 24, 48, and 72 h. Resistant – Seedlings of downy mildew resistant cultivar, Chitosan-Seedlings treated with Chitosan, *T. hamatum* UOM 13-Seedlings treated with the endophyte *T. hamatum* UOM 13, Control: Seedlings of downy mildew susceptible cultivar. Relative expression levels of (**A**) DAHP synthase (**B**) Shikimate kinase (**C**) Chorismate synthase (**D**) Chorismate mutase and (**E**) Isochorismate synthase. Expression levels were measured by qPCR and normalized to the constitutive PP2A gene. Values are means of a single experiment carried out in triplicate. The bars indicate ±SE and the data were analyzed by one-way ANOVA followed by Tukey’s test and p-value < or = 0.05 was significant compared with control and <0.01 significant with treated control.

For all the SA synthesis genes studied i.e., DAHP synthase, shikimate kinase, chorismate synthase, chorismate mutase and isochorismate synthase, constitutive levels of expression was observed in all categories of seedlings with or without pathogen inoculation, however, the level of expression was comparatively higher in resistant seedlings (Fig. 9A–E).

Expression of DAHP synthase and shikimate kinase was maximum at 12 hai in resistant, chitosan and *T. hamatum* UoM 13 treated seedlings whereas in control seedlings maximum expression was at 24 hai. In *T. hamatum* UoM 13 treated seedlings, at 12 hai expression of DAHP synthase and shikimate kinase was 3.43 and 6.40 folds higher than that of the control seedlings respectively. In the uninoculated set, *T. hamatum* UoM 13 treated seedlings at 12 h showed 2.51 and 3.21 folds higher DAHP synthase and shikimate kinase expression than the respective control. DAHP synthase expression in resistant seedlings at 12 hai was 1.19, 1.34 and 4.62 folds higher than chitosan, *T. hamatum* UoM 13 treated and control seedlings respectively. Unlike for other enzymes, expression of shikimate kinase was highest in chitosan treated seedlings compared to resistant, *T. hamatum* UoM treated and control seedlings. At 12 hai chitosan treated seedlings showed shikimate kinase expression which was 1.03, 1.09 and 6.98 folds higher than resistant, *T. hamatum* UoM treated and control seedlings respectively (Fig. 9A,B).

Maximum accumulation of chorismate synthase, chorismate mutase and IC synthase gene transcripts was observed at 24 hai for resistant, chitosan and *T. hamatum* UoM 13 treated seedlings whereas in control seedlings these expressions peaked at 48 hai. In *T. hamatum* UoM 13 treated seedlings chorismate synthase, chorismate mutase, and IC synthase expression at 24 hai was 5.50, 3.8 and 3.6 folds higher than the control seedlings respectively. In resistant seedlings, chorismate synthase expression at 24 hai was 1.41, 1.47 and 8.10 folds higher than the chitosan, *T. hamatum* UoM treated and control seedlings, respectively. Chorismate mutase expression at 24 hai in resistant seedlings was 1.21, 1.25 and 4.01 folds higher than chitosan, *T. hamatum* UoM 13 treated and control seedlings respectively. For IC synthase gene, at 24 hai the expression in resistant seedlings was on par with, 1.10 and 4.0 folds higher than chitosan, *T. hamatum* UoM treated and control seedlings respectively. In the uninoculated set, chorismate synthase, chorismate mutase, and IC synthase expression in *T. hamatum* UoM 13 treated seedlings at 24 h was 3.66, 4.32 and 4.43 folds higher than the respective controls (Fig. 9C–E).

## Discussion

Host colonization by endophytic *Trichoderma* is known to enhance plant growth and protect host plants against phytopathogens by activation of systemic acquired resistance^15^. In the present study, we showed that seed treatment with *T. hamatum* UoM 13 isolate reduced downy mildew severity on susceptible pearl millet cultivar under both greenhouse and field conditions. This was due to induction of immunity, which was both systemic and durable. The protection offered by *T. hamatum* UoM 13 seed treatment was comparable to the downy mildew protection offered by the synthetic SAR inducer chitosan and also with that of resistant check AIMP 92901-P3. The plant protective effects of *Trichoderma* species against various pathogens have been previously demonstrated in various host-pathogen interactions and particularly their role in inducing plant-mediated resistance against downy mildew of grapes and sunflower^16^,^17^. Further, *T. hamatum* species, in particular, have been very efficient in eliciting SAR in many crop plants against several pathogens^8^.

Association of *Trichoderma* strains with host roots stimulate various plant defensive mechanisms leading to SAR following pathogen infection. At the molecular level, the elicited SAR manifests in significantly enhanced production of defense metabolites like enzymes and proteins such as glucanase, peroxidase, phenylalanine ammonia lyase, and PR proteins like PR1, PR2, PR5, and PR9^18^. In our study, treatment with *T. hamatum* UoM 13 resulted in elevated levels of glucanase activity which was significantly higher in comparison to the untreated control. Glucanase is an important defense enzyme, particularly against oomycetes, as they have cellulose and glucan as major cell wall components. Our results correlate with earlier studies which have shown enhanced glucanase levels during *Trichoderma* mediated SAR in many host-pathogen systems. *Trichoderma*-induced resistance against *Phytophthora capsici* in pepper and *Trichoderma roseum*-induced resistance against *Macrophomina phaseolina* in chickpea was associated with enhanced activities of glucanase^19^,^20^.

Our results showed that *T. hamatum* UoM 13 treated seedlings recorded constitutive activity of both POX and PPO which increased after *S. graminicola* inoculation. This suggests that *T. hamatum* UoM 13 triggers POX and PPO activities which might have suppressed *S. graminicola* spreading and multiplying within the host tissues. Defense enzymes, especially POX and PPO contain the pathogen spread through the formation of polymerized phenolic barriers around the sites of infection and trigger the synthesis of anti-nutritive, antibiotic, and cytotoxic compounds leading to enhanced resistance against pathogens^21^,^22^. Several studies have emphasized the role of POX and PPO in *Trichoderma* mediated resistance in crop plants. Enhanced activities of POX, PPO, PAL and cinnamyl alcohol dehydrogenase (CAD) corresponded with *Trichoderma* species elicited resistance against, *Macrophomina phaseolina* in groundnut, *Rhizoctonia solani* seedling blight in sunflower, and *Fusarium oxysporum* f. sp. *lycopersici* in tomato^23^,^24^,^25^. This was through SAR by *Trichoderma harzianum* NBRI-1055, achieved by significantly enhanced production of defense enzymes including PAL, PPO, POX and cinnamyl alcohol dehydrogenase (CAD) activities^24^. Similarly, activities of POX and PPO were induced in significantly higher levels in tomato plants during *Trichoderma virens* induced resistance against Fusarium wilt caused by *Fusarium oxysporum* f. sp. *lycopersici*^25^.

There was a significant increase in PAL activity in *T. hamatum* UoM 13 treated pearl millet seedlings in comparison to the untreated control and The PAL activity in *T. hamatum* treated seedlings was at par with the chitosan treated and resistant pearl millet seedlings. PAL is well established as an important plant defense enzyme induced by *Trichoderma* treatment in various host-pathogen models. PAL enzyme was significantly enhanced during host resistance induction by *Trichoderma* species against *Rhizoctonia solani* in sunflower, and *F. oxysporum* and *A. alternata* in black gram^24^,^26^.

HRGPs are important structural components of plant cell walls and also accumulate in response to infection as an apparent defense mechanism. Earlier studies have indicated a role for HRGPs in pearl millet defense against oomycetes downy mildew pathogen *S. graminicola*^27^. Results of the present study indicated a 1.3 fold increase in Hyp in the cell walls of *T. hamatum* UoM 13 treated seeds compare to control. These results indicate that the seed treatment with *T. hamatum* UoM 13 triggers the defense reaction in pearl millet which includes the accumulation of HRGPs in the cell walls. The role of *Trichoderma* strains in inducing HRGPs as defensive compounds is not reported in any of the previous studies.

*Trichoderma* spp. are known to induce host resistance by reprogramming the plant proteome and^28^,^29^. *Trichoderma* spp. act upon the host tissues by invading their vascular tissue or epidermal cells of plant root leading to accumulation of signal molecules like salicylic acid (SA) and jasmonic acid (JA) which in turn result in rapid expression and up-regulation of some of the vital genes of defense enzymes/proteins^29^.

Accumulation of such defense gene transcripts generally commences within minutes to hours around the infection sites, and several hours or days later at distant sites over the whole plant. Studying the speed and magnitude of the accumulation of transcripts of these substances is very vital for devising strategies for pathogen control^30^. In the present study, *T. hamatum* UoM13 mediated systemic immunity against downy mildew, corresponded with the increased activities of several defense genes as indicated by the RT-PCR results. In general, there was a significant enhancement of activities of SA-inducible genes like glucanase, POX, PPO, PAL, HRGPs, PR-1 and PR-5 which are regarded as important SAR markers.

Transcriptomic analysis for determining the expression of defense genes and proteins in response to *Trichoderma*-induced resistance against various pathogens have been carried out in many studies, which have established that *Trichoderma* mediated resistance against phytopathogens is preceded by upregulation of genes of vital defense enzymes and PR-proteins. Transcriptional analysis of downy mildew resistance induced by *Trichoderma harzianum* T39 in susceptible grapevines showed complex transcriptional reprogramming resulting in enhanced expression of PAL, PPO, PR-1 and PR-5 genes^31^. The transcripts of glucanase and POX enzymes and PR-5 were significantly enhanced during of *Trichoderma atroviride* and *Trichoderma virens* secretory proteins mediated resistance against *Alternaria solani, Botrytis cinerea* and *Pseudomonas syringae*^32^. In tomato, *Trichoderma hamatum* 382 induced resistance against *Xanthomonas vesicatoria* in tomato and *Botrytis cinerea* in *Arabidopsis thaliana* correlated with enhanced expression of PR-5 genes^6^,^7^.

Our results are also in corroboration with earlier studies on pearl millet downy mildew system which have demonstrated that SA-inducible genes for both defense enzymes like glucanase, POX, PPO, lipoxygenase, PAL and also PR proteins like PR-1, and PR-5. These are over-expressed during SAR mediated by several biotic compounds like *Pseudomonas fluorescens, Bacillus pumilus,* and abiotic compounds like chitosan and methionine^33^,^34^,^35^,^36^.

The plant defense hormone SA is known to act as a systemic signal during induction of resistance against several pathogens^37^. Several studies have shown that SA is essential for local defense and systemic acquired resistance (SAR)^12^. Endogenous SA increased significantly upon *T. hamatum* UoM treatment which was comparable to resistant and chitosan treatments. However, the control seedlings showed delayed and lesser accumulation of SA. Further, it is to be noted that the increases in SA levels preceded the increase in various other defense enzymes and PR proteins. Our results corroborate earlier studies wherein, SA biosynthesis is significantly induced and enhanced upon challenge by a wide range of pathogens, such as tobacco mosaic virus and the bacterial phytopathogen *Pseudomonas syringae*^38^,^39^. Particularly, blocking SA accumulation significantly compromises the plant’s ability to combat biotrophic pathogens^40^.

Endogenous SA induces SAR through transcriptional reprogramming and immune responses to a broad spectrum of pathogens^37^. SA enhances expression of a several defense response genes^41^ particularly the expression of PR1 against biotrophic pathogens^42^, genes encoding β-1,3-glucanase, PR4 gene, and PR5 gene^43^,^44^.

Our results from this study show that the enzymes of SA biosynthesis pathway are up-regulated in *T. hamatum* UoM 13 treated seedlings. Increased expression of these genes occurred earlier in treated plants compared to the delayed expression in control seedlings. The genes DAHP synthase, chorismate synthase, chorismate mutase, shikimate kinase, and isochorismate synthase showed an earlier and greater expression in *T. hamatum* UoM 13 treated plants. This makes them respond faster to downy mildew infection compared to untreated plants. Such differential gene expression of SA synthesis pathway demonstrated here could possibly be linked to the ability of resistant and induced resistant (*T. hamatum* UoM 13) pearl millet plants accumulating high amounts of SA when infected by *Sclerospora graminicola*.

Various defense enzymes and signal molecules acting co-operatively may contribute to the development of an effective mechanical and chemical defense barrier in pearl millet plants against *S. graminicola* invasion. This hypothesis is substantiated by our findings showing that high levels of POX, PPO, PAL, glucanase, HRGPs, PR-1, PR-5, and SA biosynthetic gene activities in resistant and *T. hamatum* UoM 13 treated pearl millet seedlings which are correlated with high levels of resistance to downy mildew disease. The very rapid and large changes in the resistant and *T. hamatum* UoM 13 treated seedlings, in contrast to the delayed, smaller changes in the susceptible seedlings suggests that rate and magnitude of chemical defense responses are important for the effective expression of defense. This strategy may be an effective complementary option for downy mildew disease management in pearl millet.

### Materials and Methods

#### Host

Seeds of pearl millet cultivars 7042S and AIMP 92901-P3, highly susceptible and highly resistant to *S. graminicola*, respectively, were obtained from the International Crop Research Institute for Semi-Arid Tropics (ICRISAT), Hyderabad, India, and the All India Co-ordinated Research Project on Pearl Millet (AICRP-PM), Mandor, Jodhpur, India.

#### Source of pathogen and inoculum preparation

*Sclerospora graminicola* was isolated from severely infected pearl millet cv. 7042S grown under field conditions^45^. The pathogen was maintained under greenhouse conditions on its susceptible host prior to use. Leaves showing profuse sporulation of *S. graminicola* on the abaxial side were collected in the evening hours and thoroughly washed under running tap water to remove sporangia. The leaves were then blotted dry, cut into small pieces, and maintained in a moist chamber to promote sporulation. The following morning fresh sporangia were washed into distilled water. For use as inoculum, the resulting zoospore concentration was adjusted to 40,000 zoospores/ml using a hemocytometer.

#### Isolation of endophytes and inoculum preparation

Endophytic *T. hamatum* UoM 13 was isolated from root regions of twenty-day-old healthy pearl millet plants. Surface sterilization and isolation of endophyte followed a procedure as described by Hallmann *et al*. with some modifications^46^. The roots were washed thoroughly in running tap water and surface sterilized with sodium hypochlorite (2%) containing 0.1% Tween 20 for 1 min. The disinfectant was removed by rinsing the roots five times each in two washes of sterile distilled water (SDW), followed by a rinse in sterile water and then the roots were dried on sterile paper towels^47^. Roots were macerated with sterile mortar and pestle. The tissue extracts were subjected to serial dilutions. An aliquot of 0.1 ml of 10^−3^ to 10^−5^ dilutions from each of the sample was spread uniformly over the potato dextrose agar (PDA) medium supplemented with chloramphenicol (100 mg L^−1^) in Petri plates. Inoculated PDA plates were incubated at 25 ± 2 °C for 72 hours (h). After 72 h of incubation, individual fungal colonies with different morphology were picked from the edge with a sterile fine tipped needle and inoculated at the center of the PDA plates supplemented with chloramphenicol and were incubated at 25 ± 2 °C for 7 days. After incubation, pure cultures of isolated endophytic fungi were enumerated and identified individually on the basis of microscopic (conidia, fruiting body, mycelia) and macroscopic (culture morphology, color, and appearance) characteristics. Naming and classification of these fungi were made according to the standard procedures^48^.

#### Molecular identification of T. hamatum UoM 13

Morphologically identified *T. hamatum* UoM 13 was further confirmed at the molecular level by amplifying and sequencing a portion of the internal transcribed spacer (ITS) region using primers ITS-1 (TCCGTAGGTGAACCTGCGG) and ITS-4 (TCCTCCGCTTATTGATATG)^49^. A representative for each unique ITS sequence was BLAST queried to confirm species designation before being accessioned in GenBank.

#### Mass production of T. hamatum UoM 13

*Trichoderma hamatum* UoM 13 was mass multiplied on PDA plates and incubated at 25 ± 2 °C under 12/12 h alternate cycles of near ultraviolet (NUV) light and darkness for 10–12 days. After incubation, an aliquot of 10 ml of SDW was added to each of the culture plates and gently shaken to dislodge conidia from the culture surface. Conidial suspension was collected in 100 mL conical flask and passed through four layers of cheesecloth, centrifuged at 2500 rpm for 10 min and the pellet was resuspended in SDW. Conidial concentration was adjusted to 1 × 10^8^ cfu ml^−1^ using hemocytometer.

#### Seed treatment with T. hamatum UoM 13

*Trichoderma hamatum* UoM 13 was used as seed treatments. For seed treatment, 7042S seeds were surface-sterilized with 0.02% mercuric chloride for 5 min, and rinsed thoroughly in SDW. Seeds were coated with 1% gum arabic as an adhesive and suspended in the conidial suspension (5 ml/400 seeds @ 1 × 10^8^ cfu mL^−1^) and kept at 25 ± 2 °C in a rotary shaker for 6 h to ensure uniform coating. Seeds of pearl millet cultivars AIMP 92901-P3 treated with distilled water and 7042S treated with chitosan (@ 3 g kg^−1^ seed) for 3 h served as resistant and induced resistant checks respectively. 7042S seeds treated with distilled water for the same duration served as control.

#### Effect of T. hamatum UoM 13 on seed germination and seedling vigor of pearl millet under laboratory conditions

*Trichoderma hamatum* UoM 13 treated seeds and controls were seeded onto distilled water soaked brown germination paper. Fifty seeds of pearl millet were placed equidistantly on the paper. Another presoaked paper towel was placed on the first one so that the seeds were held in position. The towels were then rolled and wrapped with polythene to prevent drying. After incubation for 7 days, the towels were unrolled and the numbers of seeds germinated were counted. Seedling vigor was analyzed at the end of 7 days of incubation by the method of Abdul Baki and Anderson^50^. The length of the root and shoot of individual seedlings was measured to determine the vigor index. The vigor index was calculated using the formula: Vigor index = (mean root length + mean shoot length) × (% germination). The experiment was carried out with four replicates of 100 seeds each and was repeated three times.

#### Screening of T. hamatum UoM 13 for potential to elicit resistance against downy mildew under greenhouse and field conditions

Greenhouse studies: In the greenhouse, *T. hamatum* UoM 13 was applied as seed treatment. 7042S seeds treated with SDW and chitosan served as the control and induced resistance check respectively. 7042S seeds treated with the systemic fungicide, metalaxyl (Apron 35 SD at 6 g kg^−1^ seeds) served as fungicide treated control.

The treated seeds were sown in earthen pots filled with autoclaved soil, sand and manure at the ratio of 2:1:1. Each treatment consisted of 4 replications, ten pots per replication, and ten seedlings per pot. Treatments were arranged in a randomized complete block design. Three-day-old seedlings were challenge-inoculated by the whorl inoculation method with a zoospore suspension of *S. graminicola* at a concentration of 40,000 zoospores/ml prepared as described previously^51^. In the whorl inoculation method, droplets of *S. graminicola* zoospores were dropped onto the leaf whorl formed by the emerging seedlings and allowed to flow down to the base. These pathogen inoculated plants were maintained under greenhouse conditions (90–95% RH, 20–25 °C temperature), and observed for disease development. The plants were rated for disease when they showed any one of the typical downy mildew symptoms such as sporulation on the abaxial leaf surface, chlorosis, stunted growth, or malformation of the earheads. Downy mildew disease incidence was recorded at 30 DAS (days after sowing) and final counts were made at 60 DAS. The experiment consisted of 4 replicates of 100 seedlings each and was repeated twice.

#### Demonstration of the nature of resistance induction by T. hamatum UoM 13

This included two sets of experiments. In the first set, 7042S seeds treated with *T. hamatum* UoM 13 as described above were sown in earthen pots filled with autoclaved soil, sand and manure in the ratio 2: 1: 1. The emerging seedlings were challenge-inoculated with the zoospore suspension of *S. graminicola* by adding 4–5 drops (0.5 ml) to the leaf whorl of each plant at intervals of 1, 2, 3, 4, 5 and 6 days between the seedling emergence and pathogen inoculation in different sets of plants. In the second set 7042S seeds were plated on moist blotters and were incubated at 25 + 2 °C in an incubator. Thirty-six hours later the roots of the seedlings were inoculated with the *T. hamatum* UoM 13 by soaking the roots in the fungal spore suspension of 10^8^ cfu ml^−1^ concentration for three hours and later the seedlings were transplanted into earthen pots filled with soil, sand and manure in the ratio 2: 1:1. The seedlings were then challenge-inoculated with zoospore suspension of *S. graminicola* (40,000 zoospores/ml) following the whorl inoculation procedure with a time gap of 1, 2, 3, 4, 5 and 6 days in different sets of plants. 7042S seeds treated with distilled water was maintained as control for both the above sets of experiments. All the above sets of plants were maintained under greenhouse conditions, observed for the downy mildew disease reaction, and downy mildew disease data recorded as described earlier and the disease protection (%) was calculated as follows:


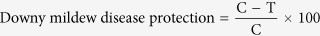


where, C, is percent downy mildew disease incidence in control; T - percent downy mildew disease incidence in treated plants.

Field studies: Field trials were conducted during rainy season 2014 and 2015 at the Indian Council of Agricultural Research (ICAR), Downy Mildew Nursery at University of Mysore in an area with soil that was heavily loaded with oospores of *S. graminicola*. The nursery is a sick plot with soil having a heavy load of *S. graminicola* oospores accumulated from the past 40 years. Oospore infested soil serves the primary source of inoculum which causes the first expression of the disease in a host population by infecting the seedlings. Oospores produced in the mature leaves of the infected plants, get mixed with the soil or seeds to initiate the disease in the next season. Secondary spread of the disease is through the airborne sporangia/zoospores of *S. graminicola. Trichoderma hamatum* UoM 13 treatments and the controls were the same as described in green house studies. Soil-borne oospores of *S. graminicola*, served as the source of primary inoculum. Additional inoculum was provided by infector rows that were raised 21 days prior to the raising of the test rows as described by Williams^52^. The experiment was a randomized complete block design and each treatment consisted of four replications and each replicated row was manually seeded with 50**–**100 seeds per row. Normal agronomic practices were followed to raise the crop. Thinning was done after 21 days to maintain a uniform number of plants per row and a uniform distance between the plants. The crop was irrigated as and when required. The plants were rated diseased when they showed any one of the typical downy mildew symptoms described above. Downy mildew disease incidence was recorded at 30 DAS, and final counts were made at 60 DAS. The experiment consisted of 4 replications and repeated twice.

### Histological, biochemical and gene expression studies

#### Plating of treated seeds

7042S seed treatments with *T. hamatum* UoM 13 and chitosan were same as described above for germination studies. In addition AIMP 92901-P3 seeds treated with distilled water served as resistant check. After treatment, the seeds were plated on pre-soaked blotters in perspex plates and incubated for two days.

#### Challenge inoculation and harvesting of seedlings

Two-day-old seedlings were root-dip inoculated with a zoospore suspension of 40,000 zoospores/ml, and incubated in dark at 25 + 2 °C. One set of the treated seedlings were inoculated with sterile distilled water which served as uninoculated control. A total of 1 g seedlings for each experiment in three replicates were harvested at 0, 3, 6, 9, 12, 24, 48 and 72 h after inoculation (hai) and immediately wrapped in aluminum foil and stored at −80 °C until further use for enzyme assays and RT-PCR analysis.

### Histological studies

#### Time course analysis of lignification

Lignification studies were carried out as described by Sherwood and Vance^53^. Pearl millet peelings were placed in 2% phloroglucinol in 95% ethanol for two hours. The tissues were then placed in a drop of 35% HCl on a slide and heated over a low-flame until the veins turned reddish purple. The slides were then observed under a Wild Leitz microscope for the intensity of coloration and the cells were counted for the lignified cells and the percentage calculated. Microscopic evaluation: in each case, 20 microscopic fields were counted for percentage calculation. The experiment was repeated five times with an average of ten plants per treatment. The peelings were examined under x500 and x1250 magnification for counting and photography respectively. A four-point scale was used to objectively assess lignification by categorizing them into 0 - no localization; + - low localization; + + - medium localization and + + + - high localization based the intensity of color.

#### Time course analysis of callose deposition

Callose deposition was studied according to the procedure of Jensen^54^. The epidermal peelings were placed in 0.005% water-soluble aniline blue in 0.15 M di-potassium phosphate for 1 h and then mounted in glycerol. They were then observed under fluorescence microscope where λ = 365–405 nm. Region with callose deposition and lignified walls fluoresced. Microscopic evaluation: in each case, 20 microscopic fields were counted for percentage calculation. The experiment was repeated five times with an average of ten plants per treatment. The peelings were examined under x500 and x1250 magnification for counting and photography respectively. A four-point scale was used to objectively assess callose deposition by categorizing them into 0 - no localization; + - low localization; + + medium localization and + + + - high localization based on fluorescence.

## Biochemical Studies

### Enzyme assays

#### Enzyme extraction

Harvested pearl millet seedlings (1 g fresh weight) were ground to a fine paste in 1 ml of buffer. The extract was centrifuged at 12,000 g for 20 min at 4 °C and the supernatant was transferred to a new tube and used as the enzyme extract.

#### Protein estimation – Lowry’s method

To calculate the specific activity of the enzymes, protein content in the crude extract was estimated by Lowry’s method using BSA (Sigma) as a standard^55^.

#### Phenylalanine ammonia-lyase assay

PAL enzyme was extracted with 25 mM Tris HCl buffer (pH 8.8). PAL activity was assayed according to the procedure of Beaudoin-Eagan and Thorpe^56^. One hundred microlitres of extracts were mixed with 900 ml of 50 mM L-Phenylalanine and 100 mM Tris HCl buffer solution (pH 8.01). The mixture was placed in a water bath at 40 °C for 120 min. The reaction was stopped by adding 60 ml of 5 N HCl. Enzyme activity was determined as the amount of t-cinnamic acid formed from L-Phenylalanine per mg of protein per min measured spectrophotometrically at a wavelength of 290 nm.

#### Peroxidase assay

POX enzyme was extracted in 10 mM potassium phosphate buffer (pH 6.9). POX activity was assayed according to the procedure of Hammerschmidt *et al*.^57^. The reaction mixture (3 ml) consisted of 0.25% v/v guaiacol in 10 mM potassium phosphate buffer (pH 6.0) containing 100 mM hydrogen peroxidase. The crude enzyme (10 mL) was added to initiate the reaction, which was measured spectrophotometrically at 470 nm. One unit of POX enzyme activity is defined as the increase in absorbance recorded 470 nm. POX activity is expressed in terms of the change in A_470_ min^−1^ mg^−1^ protein.

#### β-1,3-Glucanase assay

β -1,3-Glucanase enzyme was extracted with 50 mM sodium acetate buffer (pH 5.2). β-1,3-Glucanase activity was assayed according to the method of Pan *et al*.^58^, with glucose as standard. 0.1% Laminarin (Sigma) in 0.05 M sodium acetate buffer (pH 5.2) was used as the substrate and 50 ml enzyme as extract and incubated for 15 min at 37 °C. The reaction was stopped by adding 0.5 ml of DNS reagent, incubated in boiling water bath for 10 min and cooled and finally 1 ml distilled water was added. Products released after incubation were estimated for reducing groups at 540 nm. Enzyme activity was expressed in terms of μ moles per mg per min.

#### Polyphenol oxidase assay

PPO enzyme was extracted in Tris–HCL buffer (pH 7.0) containing 0.1 M KCl, 1% (v/v) TritonX-100,1 mM EDTA and 5% (w/v) Polyvinylpolypyrrolidone (PVPP). The reaction mixture (3 ml) consisted of 10 mM catechol in 100 mM potassium phosphate buffer (pH 6.5) was assayed as described by Arora and Bajaj^59^. The standard reaction mixture consisted of 3 ml of 10 mM sublimated catechol in 100 mM potassium phosphate buffer (pH 6.5) and 10 ml of enzyme extract. Increase in absorbance at 420 nm was recorded for 1 min. The results are expressed as the change in A per min per mg protein.

### Analysis of Hydroxyproline-rich glycoproteins

Cell walls from the pearl millet seedlings were obtained by modifying the procedure of York *et al*.^60^. The seedlings were homogenised using pestle and mortar at 4 °C in 0.5 M potassium phosphate buffer, pH 7.0. The paste was observed under microscope for complete disruption of cells. The suspension of broken cells was centrifuged at 2000 × *g* for 10 min. Cell walls were repeatedly washed with the above buffer followed by distilled water. Washed cell walls were suspended by vigorous stirring in 5 volume of 1:1 chloroform–methanol. The organic solvent was carefully removed. Cell walls were repeatedly washed with 5 volume of acetone and then air dried. The amount of HRGPs was determined by analyzing the Hyp content in the cell wall hydrolysate.

Cell walls were hydrolyzed in sealed tubes with 6 N HCl for 18 h at 110 °C. To remove HCl, the hydrolysates were evaporated to dryness. Hydroxyproline was then extracted in minimum amount of distilled water from the dried hydrolyzed samples and determined following the spectrophotometric method of Prockop and Udenfriend^61^. Hyp content was expressed as μg Hyp mg^−1^ cell wall (dry weight).

### Analysis of endogenous Salicylic acid accumulation

#### Partial purification of SA from pearl millet seedlings

Tissue samples of one gram were ground in 1.0 ml of 90% methanol using a mortar and pestle. The extract was centrifuged at 10,000 rpm for 15 min at 40 C. The pellet was re-suspended in 100% methanol, re-extracted and centrifuged as above. Supernatants from both extractions were combined and dried in vacuo. Further steps in the purification of the sample were followed according to the method described by Raskin *et al*.^62^. The samples were analyzed for free SA after acid hydrolysis.

#### High performance liquid chromatography

Standard graph and SA quantification in test samples was carried out by following the procedure of Meuwly and Metraux^63^. Reverse phase HPLC was performed on a Shimadzu LC 10 AS equipped with a 5 μm particle size C 18 column [4.6 × 150] maintained at 210 C. For the preparation of the standard graph, a stock of 1 mg ml^−1^ of commercial grade SA was prepared in the mobile phase (7:3 methanol/water), diluted to different concentrations ranging from 1–10 ng and 10 μl of each concentrations was directly injected and the flow rate was maintained at 1 ml min^−1^. Standard graph plotted with concentration of SA on X-axis and peak area on Y-axis was used for quantifying the amount of SA in test samples. Wavelength scan identified an elicitation wavelength of 312 nm and an emission wavelength of 415 nm for optimum quantification of SA.

### Gene expression studies

#### Quantitative real-time PCR analysis (qPCR) for defense enzymes, hydroxyproline-rich glycoproteins, pathogenesis-related proteins, and SA synthesis genes

RNA extraction: A total of 100 mg of frozen seedlings was ground to fine powder in a 2 ml SealRite microcentrifuge tube using stainless steel beads and an automated shaker SO-10M (Fluid Management, Wheeling, IL, USA). Total RNA was extracted from seedlings harvested at different times noted above by using the RNeasy plant mini Kit (Qiagen,) as per the manufacturer’s instructions. Eluted RNA was stored at −80 °C and then treated with DNase I (RNase free) (Fermentas). The concentration and purity of RNA was determined by means of spectrophotometer and its integrity by agarose gel electrophoresis.

RT-PCR analysis: The relative quantitation of PAL (NM001174615.1), POX (EU492461), β -1,3-Glucanase (EU725041.1), PPO (AY881993.1), HRGP (GQ223398), PR1 (HQ699781.1), PR5 (EU725133.1), DAHP synthase (M64261), Chorismate mutase (AB182997), Isochorismate synthase (AY740529), Chorismate synthase (EB427412), Shikimate kinase (EB425065), *mRNAs* in pearl millet seedlings was done by using gene-specific primers (Eddo, Alexander, 2008), designed with Primer Express version 3.0 software (Applied Biosystems) (Table 2). PP2A (protein phosphatase 2 A) served as endogeneous reference gene. Primer specificities were confirmed by agarose gel electrophoresis of the RT-PCR products. Each qPCR reaction (20 μL) consisted of 1× SYBR Green PCR master mix (SYBR Green mix, Applied Biosystems), 3 pmol of each primer and 20 ng each of cDNA and used StepOnePlus™ Real-Time PCR Systems (Applied Biosystems). qPCR steps were: denaturation at 95 °C for 10 min, 40 cycles of 15 s at 95 °C, 60 s at 60 °C. At the end of each reaction, a melting curve was created using a single cycle consisting of 15 s at 95 °C and 60 s at 60 °C. This was followed by a slow temperature increase to 95 °C at the rate of 0.3 °C s^−1^. The quantification of target mRNAs used a comparative Ct method^64^.

### Data analysis

Data from greenhouse and field experiments were analyzed separately for each experiment and were subjected to arcsine transformation and analysis of variance (JMP Software; SAS Institute Inc., Cary, NC). Significance effects of treatments were determined by the magnitude of the F value (P = 0.05). Treatment means were separated by Tukey’s honest significant difference test.

## Additional Information

**How to cite this article**: Siddaiah, C. N. *et al*. Elicitation of resistance and associated defense responses in *Trichoderma hamatum* induced protection against pearl millet downy mildew pathogen. *Sci. Rep.* **7**, 43991; doi: 10.1038/srep43991 (2017).

**Publisher's note:** Springer Nature remains neutral with regard to jurisdictional claims in published maps and institutional affiliations.

## Supplementary Material

Supplementary Information

## Figures and Tables

**Table 1:** Effect of *T. hamatum* UoM 13 treatment on pearl millet seed germination, seedling vigour and downy mildew disease incidence.

Treatment	Concentration	Germination (%)	Seedling vigor	Downy Mildew Incidence (%)	
				Green house	Field
*T. hamatum* UoM 13	1 × 10^8^ cfu ml^−1^	94 ± 2.08^a^	1928 ± 8.62^a^	18.2 ± 0.41^b^	15.6 ± 0.32^b^
Chitosan	3 g kg^−1^	90 ± 2.30^ab^	1907 ± 10.11^ab^	17.4 ± 0.66^b^	12.5 ± 0.41^b^
Apron 35 SD	6 gm/kg of seeds	88 ± 1.73^ab^	1896 ± 18.02^ab^	8.8 ± 0.51^c^	5.1 ± 0.46^c^
Control	Untreated	83 ± 2.64^b^	1872 ± 9.64^b^	96 ± 1.73^a^	92.5 ± 2.29^a^

Percentages of seed germination and vigour index are mean from three repeated experiments. Vigour index was calculated on percentage germination and mean root and shoot lengths of the seedlings. The values are mean from three experiments. Means designated with the same letter in column are not significantly different according to Tukey’s HSD test at P = 0.05.

**Table 2:** Primer sequences used for qRT-PCR amplification.

Sl. No.	Target gene amplified	Forward primer sequence (5’ to 3’)	Reverse primer sequence (5’ to 3’)
1.	Phenyl alanine ammonia lyase	ATGGAGTGCGAGAACGGCC	CTGCGCGATGCTGAGGCT
2.	Peroxidase	CCCCAGAAGCACATTTGTGA	CATGGCTGCGGGCGGAG
3.	β -1,3-Glucanase	ATGGCGAGGCAGGGTGTCATC	GGATTGGACTCCTGTTTA
4.	Polyphenol oxidase	AGTCGAGGTTTGGCCACCAT	CCACCTGATGCGCTCGATG
5.	PR1	TGGACGTGCCGCTGCCG	GAACTGCGCCGCCACACG
6.	PR-5	GCGTCCTCGGTCCTCCTG	CACACGCGGCCGGAGCTG
7.	Hydorxy rich glyco proteins	GCCTAAGCCGAAGCCACCAA	GCGTGTAGGTCGGAGGAGTT
8.	DAHP Synthase	GGC TCA ATT TCA GGT ACC	GTT AGA GTC GGT AAG TAA
9.	Shikimate Kinase	CTC ACC TAC CTC TCT CTC A	AAG GCT CTG CGA AAC TCT
10.	Chorismate Synthase	ATC TTC CAA TCT TCA TATA	TCC TAG GTG TGG TAA TTC
11.	Chorismate Mutase	CTT CAA TCT AAG GTT GGT AG	CTG AAT ATC ACA GGA AGC AG
12.	IC Synthase	CAG GTT GAG TTT GAT GAG CT	CTT GAT AAG CAT CGG GTT
Reference housekeeping gene			
1.	Protein phosphatase 2A	TGAGAGCAGACAAATCACTCAA	AAGAGCTGTGAGAGGCAAATAA
