# Supplementary material for: Elicitation of resistance and associated defense responses in Trichoderma hamatum induced protection against pearl millet downy mildew pathogen
Source: Sci Rep. 2017 Mar 21;7:43991. doi: 10.1038/srep43991 (PMC5359564; doi:10.1038/srep43991)
Supplement: Supplementary Information [file srep43991-s1.doc]

**Elicitation of resistance and associated defense responses in *Trichoderma hamatum* induced protection against pearl millet downy mildew pathogen**

Chandra Nayaka Siddaiah^1*^, Niranjan Raj Satyanarayana^2^, Venkataramana Mudili^3^, Vijai Kumar Gupta^4^, Selvakumar Gurunathan^3^, Shobith Rangappa^5^, Shekar Shetty Huntrike^1^, Rakesh Kumar Srivastava^6^*

^1^Department of Studies in Biotechnology, University of Mysore, Manasagangotri, Mysore-570006, Karnataka-, India.

^2^Department of Studies in Microbiology, Karnataka State Open University, Mukthagangotri, Mysore – 570006, Karnataka, India.

^3^Microbiology Division, DRDO-BU-Centre for Life sciences, Bharathiar University Campus, Coimbatore-641046, Tamil Nadu, India.

^4^Discipline of Biochemistry, National University of Ireland Galway, Galway, Ireland

^5^Frontier Research Center for Post-Genome Science and Technology, Hokkaido University, Sapporo 060-0808, Japan.

^6^International Crops Research Institute for the Semi-Arid Tropics (ICRISAT), Patancheru-502324, Telangana, India.


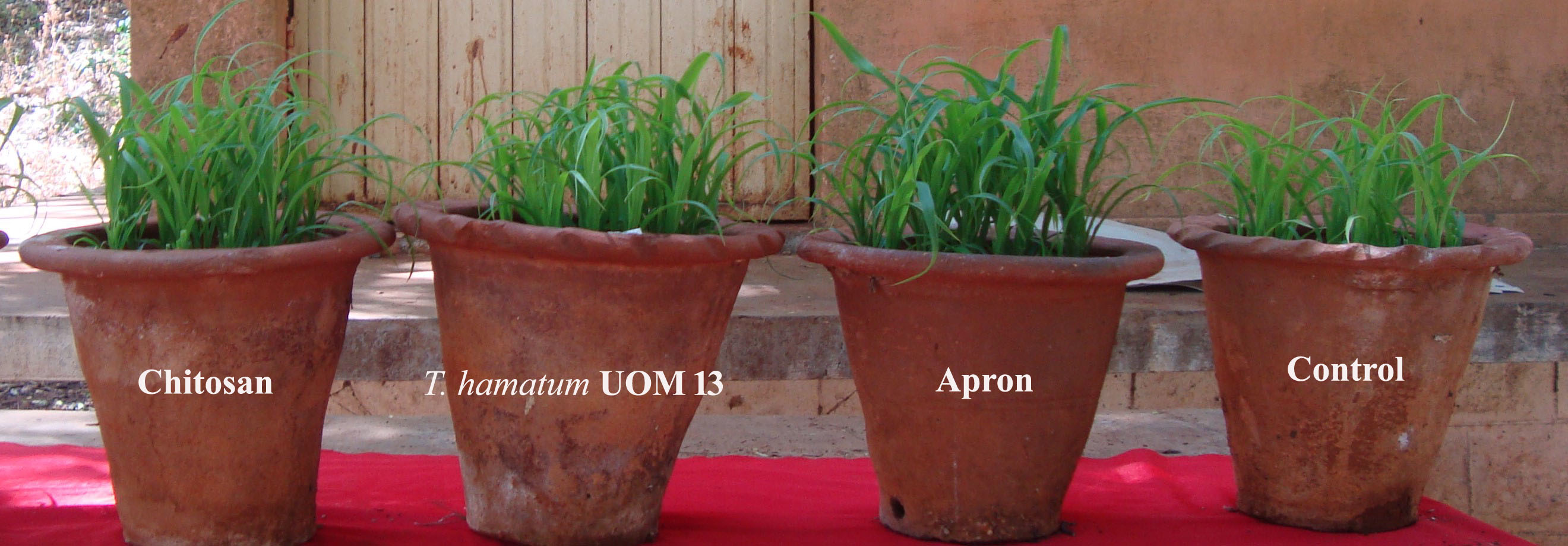


**Figure 1:** Illustration of the effect of pearl millet seed treatment with Chitosan, *T. hamatum* UOM 13, Apron on downy mildew disease incidence in comparison to the untreated control plants under greenhouse conditions. Pearl millet seeds cultivar 7042S which is highly susceptible to downy mildew were treated with Chitosan and *T. hamatum*, seeds of the cultivar AIMP 92901-P3 was used as resistant check, and 7042S seeds without any treatment was the susceptible control. The seeds were later sown in earthen pots and the emerging two-day-old seedlings were whorl inoculated with zoospores of Sclerospora graminicola and observed for downy mildew disease symptoms.

**
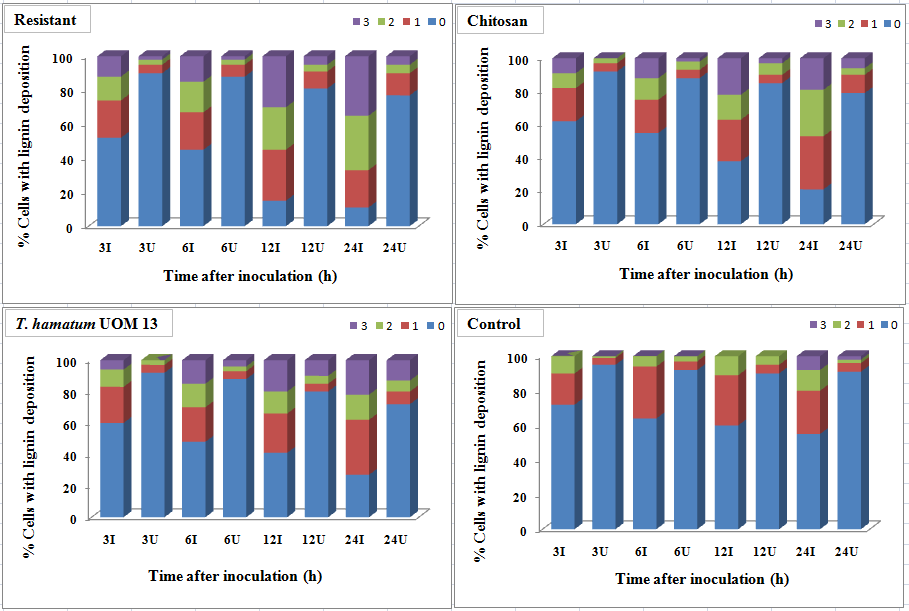
**

**Figure 2:** Temporal pattern of the degree of lignification observed in cells of the epidermal peelings of coleoptile regions of pearl millet seedlings with (I) or without (U) *Sclerospora graminicola* inoculation. Resistant – Seedlings of downy mildew resistant cultivar, Chitosan-Seedlings treated with Chitosan, *T. hamatum* UOM 13-Seedlings treated with the endophyte *T. hamatum* UOM 13, Control: Seedlings of downy mildew susceptible cultivar. Results are average of three independent experiments with four replicates of twenty five seedlings each. 0 – no deposition, 1 – low deposition, 2 – medium deposition, 3 – high deposition.

**
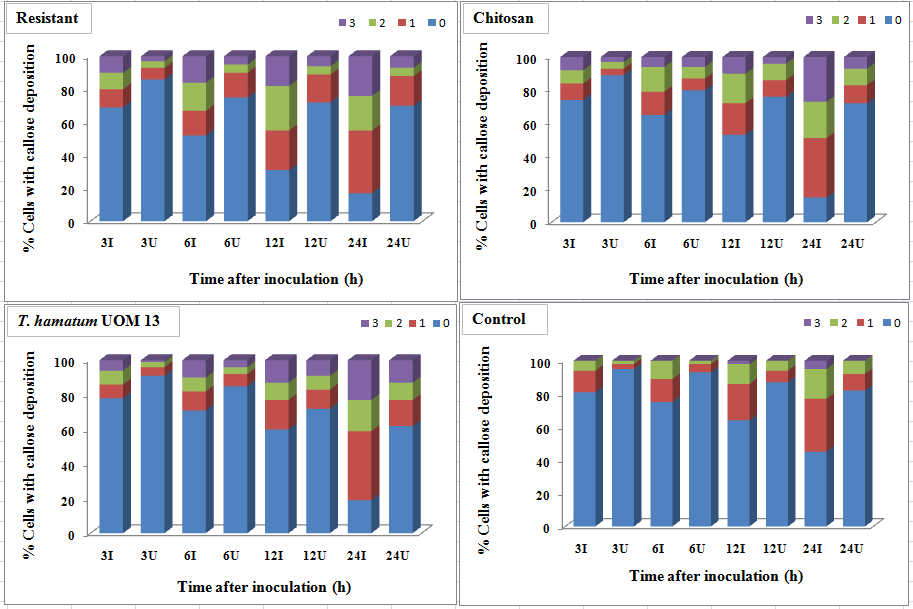
**

**Figure 3:** Temporal pattern of the degree of callose deposition observed in cells of the epidermal peelings of coleoptile regions of pearl millet seedlings with (I) or without (U) *Sclerospora graminicola* inoculation. Resistant – Seedlings of downy mildew resistant cultivar, Chitosan-Seedlings treated with Chitosan, *T. hamatum* UOM 13-Seedlings treated with the endophyte *T. hamatum* UOM 13, Control: Seedlings of downy mildew susceptible cultivar. Results are average of three independent experiments with four replicates of twenty five seedlings each. 0 – no deposition, 1 – low deposition, 2 – medium deposition, 3 – high deposition.

**
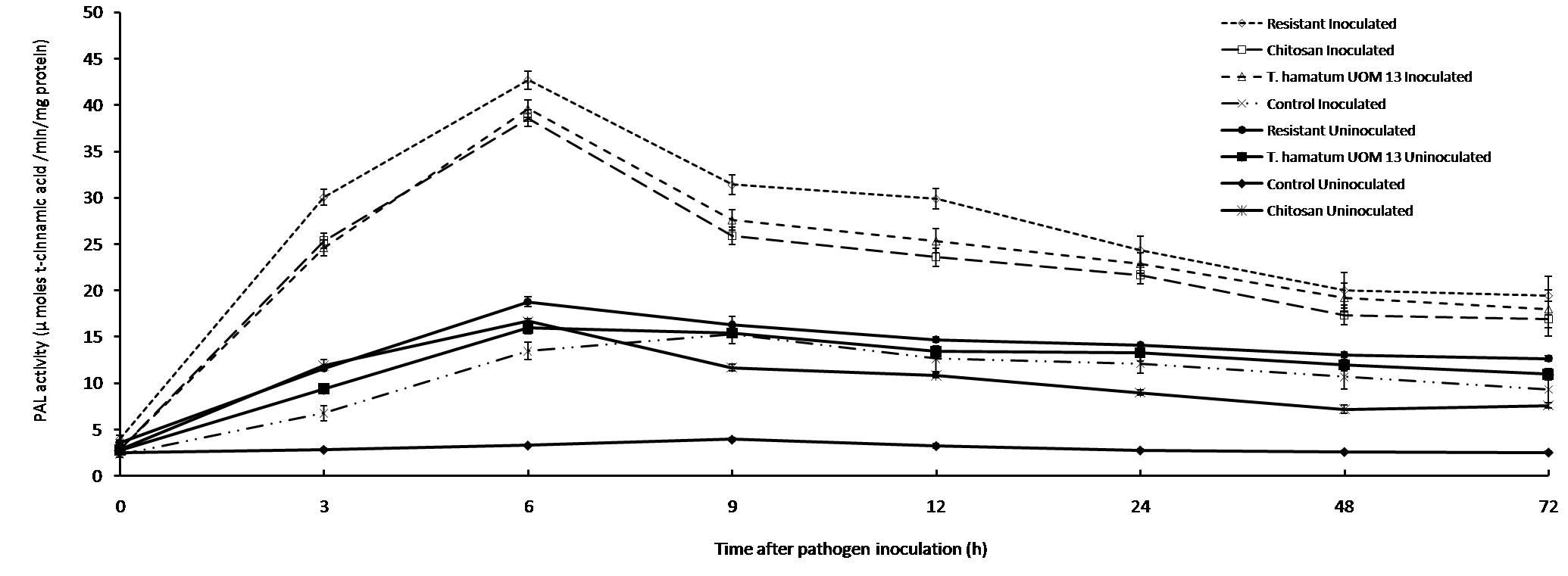
**

**A**

**
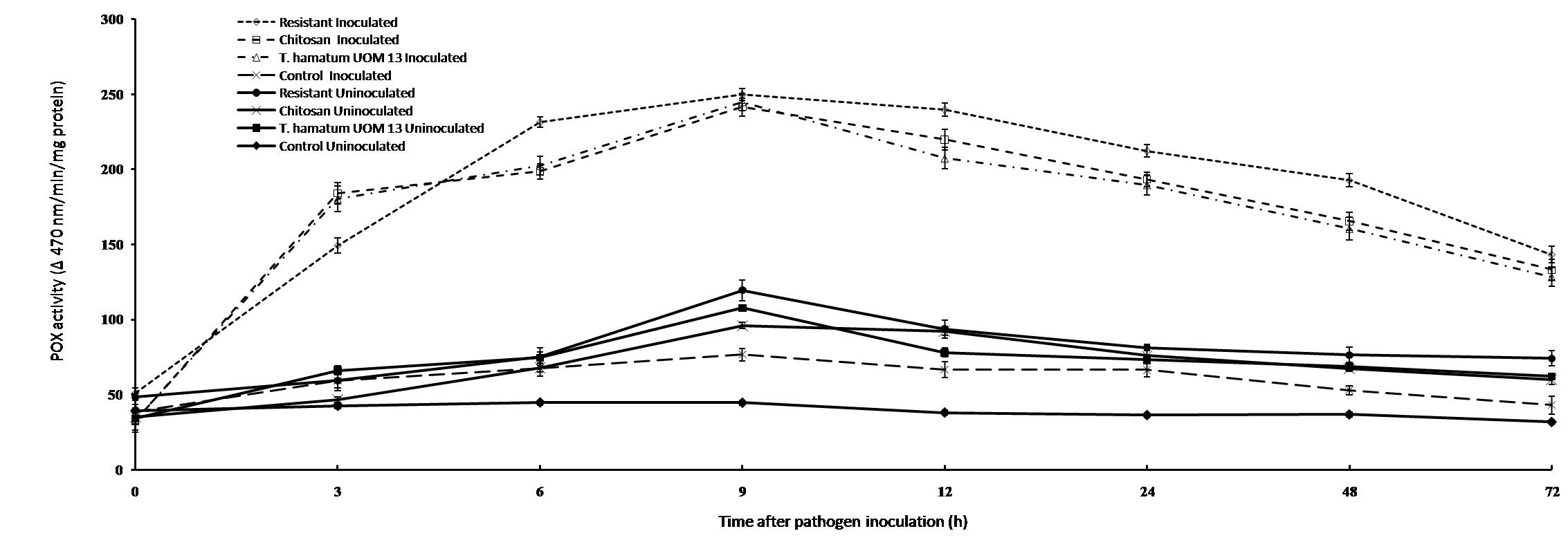
**

**B**

**
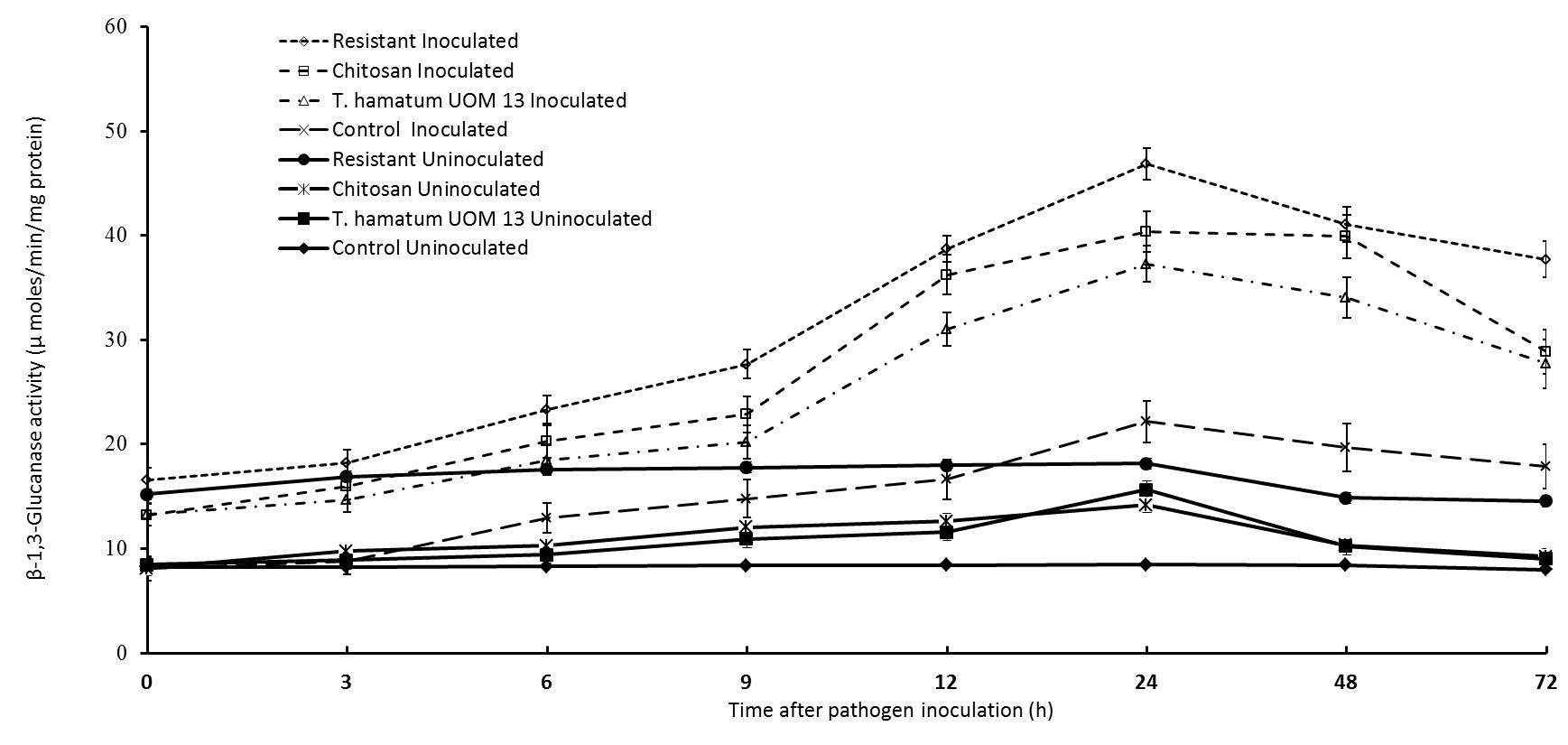
**

**C**

**
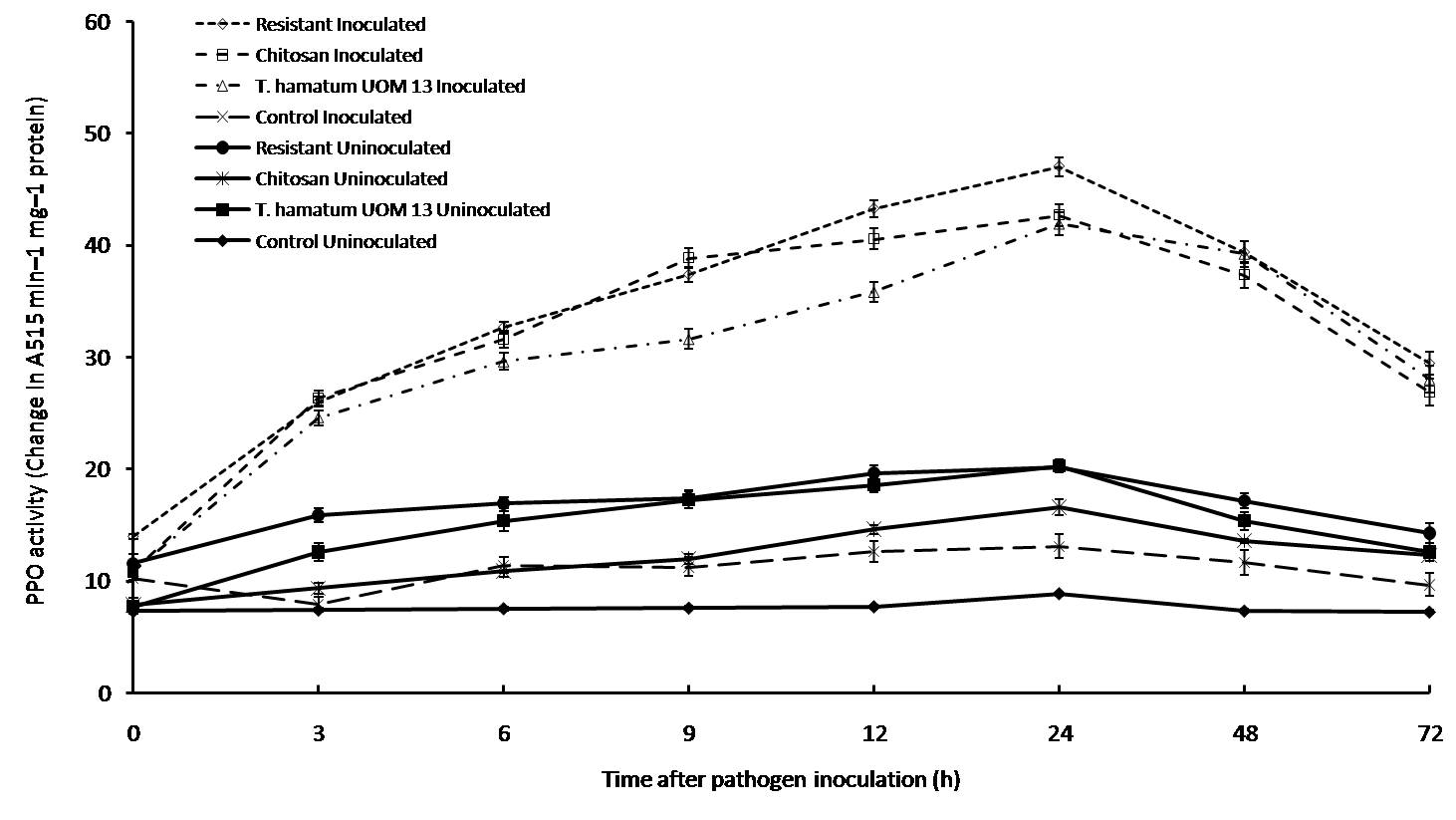
**

**D**

**Figure 4:** Temporal pattern of accumulation of defense-related enzymes in two-day old pearl millet seedlings harvested at 0, 3, 6, 9, 12, 24, 48 and 72 h with (inoculated) or without (uninoculated) *Sclerospora graminicola* inoculation. Resistant – Seedlings of downy mildew resistant cultivar, Chitosan-Seedlings treated with Chitosan, *T. hamatum* UOM 13-Seedlings treated with the endophyte *T. hamatum* UOM 13, Control: Seedlings of downy mildew susceptible cultivar. (A) Phenylalanine ammonia lyase was determined as activity was determined as the amount of t-cinnamic acid formed from L-Phenylalanine per mg of protein per min measured spectrophotometrically at a wavelength of 290 nm (B) Peroxidase activity determined as the increase in absorbance recorded 470 nm. POX activity is expressed in terms of the change in A_470_ min^−1^ mg^−1^ protein (C) β-1,3 Glucanase activity was expressed in terms of µ moles per mg protein per min (D) Polyphenol oxidase determined as increase in absorbance at 420 nm was recorded for 1 min. The results are expressed as the change in A per min per mg protein Data of enzyme activity are means ± SE of three different experiments. The values were the means of three replicates of three different experiments. Bars indicate standard errors; means with different superscripts are significantly different, as shown by Tukey’s HSD test (P = 0.05)
